# Phage as a prospective arsenal against superbugs

**DOI:** 10.1007/s11033-026-12537-9

**Published:** 2026-08-04

**Authors:** Lejia Zhao, Yirui Wan, Sharon Shui Yee Leung, Xuemei Yang, Ping Zeng

**Affiliations:** 1 The State Key Laboratory of Pharmaceutical Biotechnology, School of Life Sciences, Nanjing University, Nanjing, 210023 Jiangsu China; 2 Department of Laboratory Medicine, Nanjing Drum Tower Hospital, Nanjing University Medical School, Nanjing, 210008 Jiangsu China; 3 School of Pharmacy, Faculty of Medicine, The Chinese University of Hong Kong, Shatin, 999077 Hong Kong China; 4 College of Biological and Chemical Engineering, Qilu Institute of Technology, Jinan, 250200 China

**Keywords:** Bacteriophage, Phage therapy, Antimicrobial resistance, Phage engineering, Clinical translation

## Abstract

The escalating crisis of antimicrobial resistance (AMR) necessitates the urgent development of alternatives to traditional antibiotics. Bacteriophage (phage) therapy, which utilizes viruses that specifically infect and lyse bacteria, has re-emerged as a promising therapeutic strategy. This review comprehensively examines the current landscape, beginning with the modern, genome-based classification of phages and detailing their key therapeutic advantages, including high specificity, self-amplification, and biofilm-penetrating capability. We explore advanced biotechnological applications such as genetic engineering of phages, the use of phage-derived proteins, and synergistic phage-antibiotic combinations (PAS). The translational workflow from phage sourcing and biobanking to characterization, formulation, and clinical delivery is critically analyzed, alongside major therapeutic areas like chronic wounds and pulmonary infections. Despite promising clinical evidence from compassionate use and trials, significant scientific, regulatory, and commercial hurdles remain. The integration of synthetic biology and artificial intelligence is poised to overcome these challenges, steering phage therapy toward becoming a precise and adaptable component of the modern antimicrobial arsenal.

## Introduction

The rapid global escalation of antimicrobial resistance (AMR) is currently one of the most critical public health challenges. It is estimated that AMR is directly responsible for millions of infections and contributes to over a million deaths annually, a figure projected to grow exponentially in the coming decades [1]. However, the pipeline for novel antibiotics has dwindled, failing to keep pace with the evolution of resistant pathogens and leaving clinicians with fewer effective therapeutic options. Consequently, it is urgent to look beyond conventional antibiotics and develop alternative antimicrobial strategies. Bacteriophages (or phages), viruses that specifically infect and lyse bacterial cells, have re-emerged as a promising therapeutic alternative [2]. Their natural role as ubiquitous and potent bacterial predators underscores a time-tested, co-evolutionary strategy for bacteria control which can be harnessed for clinical applications [3].

The concept of phage therapy is not new, but it is undergoing a significant resurgence. Its renewed appeal is driven by its unique therapeutic advantages, including high target specificity, efficacy against both antibiotic-sensitive and antibiotic-resistant pathogens, and limited direct toxicity to human cells when appropriately selected and purified [4]. Furthermore, the vast natural reservoir of bacteriophages provides a rich source for therapeutic discovery, and their potential for synergistic use with existing antibiotics enhances their clinical promise. However, translating this potential into standardized, widely available treatments confronts considerable scientific and commercial hurdles. Unlike broad-spectrum antibiotics, phages typically exhibit a narrow host range, which complicates presumptive or empiric treatment strategies [5]. Significant regulatory pathways for phage-based biologics are still under development, and the commercial landscape is challenged by intellectual property issues and a historically fragmented development model [6]. This review seeks to explore the current landscape of phage therapy, examining its potential to address the AMR crisis while critically analyzing the barriers that must be overcome for its successful integration into modern medicine.

## Bacteriophage biology and mechanisms of action

### Structure, classification, and how it works

**Fig. 1:**
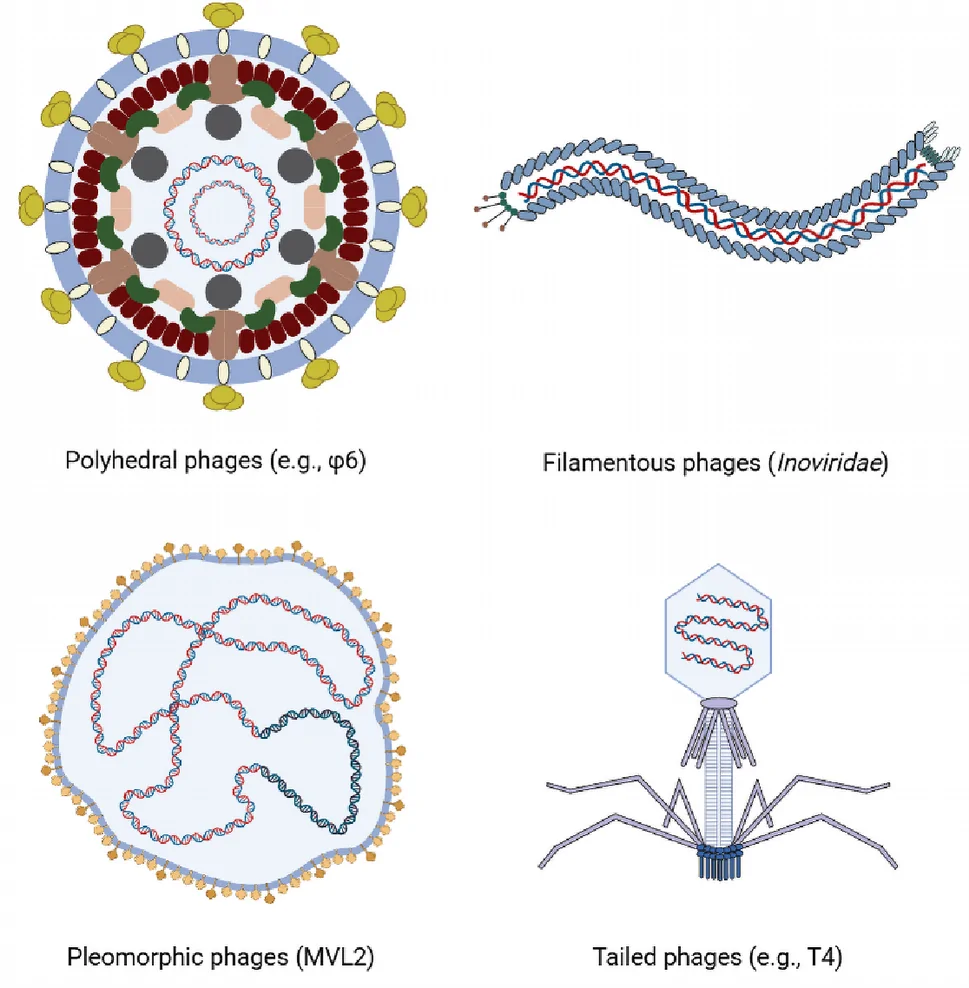
Representative structures of bacteriophages. Bacteriophages have diverse structures, including enveloped polyhedral, filamentous, pleomorphic, and tailed forms. Phage φ6 contains a segmented double-stranded RNA genome enclosed by a multilayered capsid and lipid envelope. Filamentous phages of the family *Inoviridae* have a long helical protein coat surrounding a single-stranded DNA genome. MVL2 is a pleomorphic, enveloped phage containing a nucleoprotein complex. T4 has a prolate icosahedral head, a contractile tail, a baseplate, and long tail fibers. The tail fibers are involved in host recognition and attachment, while the tail structure helps deliver the genome into the bacterial cell. The structures are schematic and not drawn to scale

Bacteriophages exhibit remarkable diversity in size, morphology, and genomic organization. Their fundamental architecture consists of a nucleic acid genome (DNA or RNA) encased in a protective protein shell, the capsid (Fig. 1**)** [7]. The capsid protects the genetic material, while the tail structure facilitates its delivery into the host cell.

**Fig. 2:**
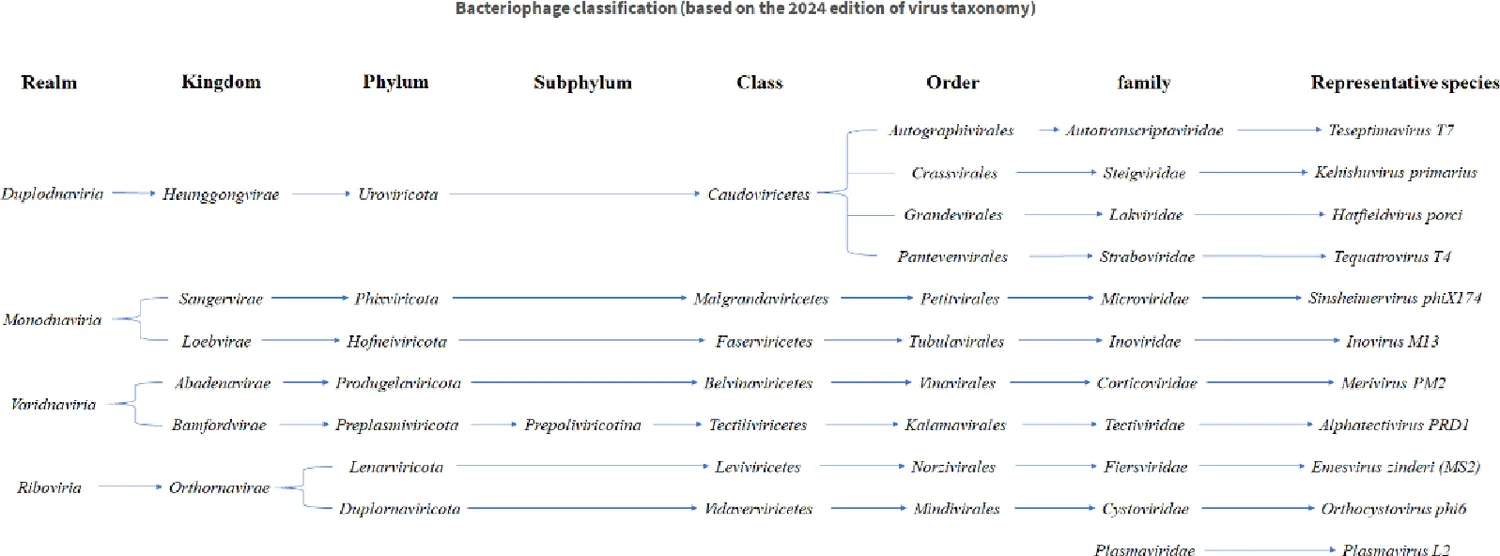
Current broad classification of bacteriophages. The figure shows representative bacteriophage taxa from realm to species. The class Caudoviricetes comprises 11 orders, of which four bacterial virus orders are presented with representative families and species. The former family Leviviridae has been reorganized within the class Leviviricetes, including the family Fiersviridae. Only selected lineages are shown

The formal classification of bacteriophages is governed by the International Committee on Taxonomy of Viruses (ICTV) and is currently undergoing a significant transition (Fig. 2) [8]. The field is moving from a system based on phenotypic characteristics like morphology and host range toward a genome-centered phylogenetic framework. Historically, phage taxonomy relied largely on genome type (dsDNA, ssDNA, dsRNA, ssRNA) and virion morphology [9]. Based on virion morphology, phages are broadly categorized into four major types: polyhedral phages (*Microviridae*, *Corticoviridae*, *Tectiviridae*, *Leviviridae*, and *Cystoviridae*), filamentous phages (*Inoviridae*), pleomorphic phages (*Plasmaviridae*), and tailed phages (*Caudovirales*). In the current ICTV system, the traditional order *Caudovirales* and its three major morphological families, *Myoviridae*, *Siphoviridae*, and *Podoviridae*, no longer hold official taxonomic status [10]. This restructuring reflects evidence from modern genome-based phylogenetic analyses showing that morphology-based groupings were not monophyletic. Following this revision, ICTV established the class *Caudoviricetes* to encompass all tailed bacterial and archaeal viruses with double-stranded DNA genomes. Within current phage taxonomy, dsDNA tailed phages constitute the most common and abundant group [11]. They exemplify the canonical bacteriophage architecture, with a dsDNA genome packaged within an icosahedral capsid (head) attached to a tail that mediates host recognition and genome delivery (Fig. 1).

**Fig. 3:**
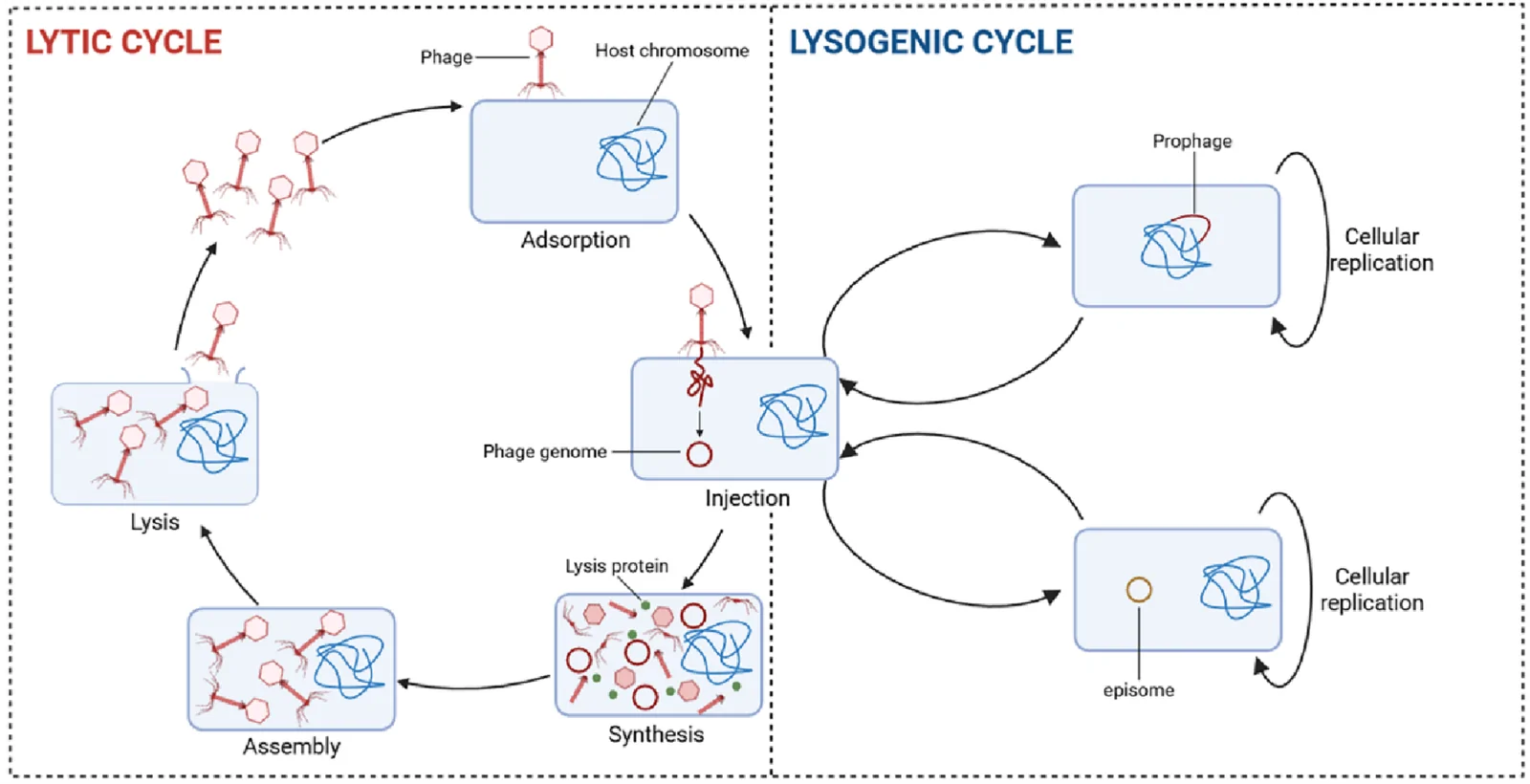
Life cycles of bacteriophages. The lytic cycle includes adsorption, genome injection, synthesis, assembly, and host-cell lysis. In the lysogenic cycle, the phage genome integrates into the host chromosome or remains as an episome and replicates with the host cell. Under certain conditions, it may enter the lytic cycle. The illustration is schematic and not drawn to scale

Bacteriophages follow either a lytic or lysogenic life cycle (Fig. 3). The lytic cycle is a destructive, five-stage process that results in host cell death and the release of new virions [12]. This process begins with adsorption, during which the phage uses tail structures such as tail fibers or tail spikes to precisely recognize and bind specific receptors on the bacterial surface. This is followed by injection, during which the phage genome is delivered into the cytoplasm, often accompanied by contraction of the tail sheath in contractile-tailed phages. Once inside the cell, the phage enters the synthesis stage, hijacking host cellular machinery to replicate its own genome and produce structural components and lysis proteins. During assembly, newly synthesized genomes are packaged into procapsids through the portal complex, and subsequently joined with tail structures to form mature progeny virions. Finally, in the lysis stage, the phage expresses holins and endolysins, as well as spanins in Gram-negative hosts. These factors disrupt the cell membrane and peptidoglycan layers, causing host cell lysis and the release of hundreds of progeny virions to initiate new rounds of infection. In the lysogenic pathway, following injection, the viral genome, known as a prophage, integrates into the host chromosome or persists as an episome, replicating passively with the host cell [13]. Under stress conditions, the prophage can exit lysogeny, activate lytic gene expression, assemble viral particles, and eventually be released from the host cell through lysis. Lytic phages infect bacteria predominantly through the lytic pathway. Temperate phages, by contrast, may enter either the lytic or lysogenic cycle. Lytic phages are preferred in clinical applications due to their reliable, direct lethality [14]. In contrast, temperate phages are generally excluded from therapeutic development because they can enter lysogeny, promoting host survival and posing a risk of horizontal transfer of virulence or antibiotic resistance genes [15].

### Key therapeutic advantages and limitations

#### Target specificity and microbiome-related considerations

A major potential advantage of bacteriophages is their target specificity, which often results in a narrower antibacterial spectrum than that of conventional broad-spectrum antibiotics. Phage infection is initiated through interactions between receptor-binding proteins (RBPs), typically located on tail fibers, tail spikes, or other adsorption structures, and compatible receptors on the bacterial surface [16]. These receptors may include capsular polysaccharides, lipopolysaccharides, outer membrane proteins, or other cell surface structures. Productive infection, however, depends not only on receptor recognition and adsorption but also on successful genome entry, evasion of intracellular bacterial defense systems, replication, assembly, and progeny release.

Compared with broad-spectrum antibiotics, phages with a narrow host range may cause less collateral damage to commensal bacteria. This may be especially beneficial during prolonged treatment or when preserving the intestinal microbiome is clinically important. Targeted phage therapy may therefore help preserve commensal bacteria and reduce the risk of dysbiosis-related complications [17]. However, direct clinical evidence that phage therapy consistently preserves the microbiome or prevents dysbiosis-related complications remains limited.

Moreover, narrow host specificity represents both an advantage and a therapeutic limitation. Phages that are active against one clinical isolate may be ineffective against other strains of the same bacterial species because of variation in surface receptors, capsules, restriction-modification systems, CRISPR–Cas systems, and other antiphage defenses. Therefore, effective treatment usually requires accurate pathogen identification and isolate-level phage susceptibility testing. Phage cocktails can broaden bacterial coverage and reduce the risk of rapid resistance emergence, but they also increase formulation complexity and make manufacturing, quality control, and pharmacokinetic and pharmacodynamic evaluation more challenging.

Although phages do not target non-susceptible bacteria, selective removal of particular bacterial populations may indirectly disrupt microbial community balance. Thus, microbiome preservation should be regarded as a potential benefit that depends on the treatment context, rather than a guaranteed outcome of phage therapy.

#### Self-amplification and self-limiting pharmacodynamics

Lytic phages differ from conventional antimicrobial agents because they can replicate in susceptible bacteria and increase in number at the infection site. Under suitable conditions, phages can infect target bacteria, produce progeny, and initiate new infection cycles, thereby increasing local antibacterial activity without a proportional increase in the administered dose [18]. This property may be particularly useful when the infection site contains enough metabolically active and phage-susceptible bacteria.

However, effective amplification in vivo is not guaranteed. It depends on bacterial density and physiological state, phage replication ability, receptor accessibility, tissue penetration, bacterial antiphage defenses, and host immune clearance. Biofilms, poorly vascularized tissues, low bacterial metabolic activity, and the emergence of phage-resistant populations may limit productive infection. Administered phages may be rapidly cleared by the mononuclear phagocyte system or neutralized by anti-phage antibodies, limiting their concentration at the infection site [19].

As the number of susceptible bacteria decreases, phage replication also declines. However, phages may remain in the body after replication has stopped, and their clearance depends on phage properties, formulation, administration route, tissue distribution, and host immune responses. Therefore, phage self-amplification and self-limitation depend on the treatment conditions. Phage-specific pharmacokinetic and pharmacodynamic models are needed to guide dose selection and treatment monitoring.

#### Biofilm penetration and eradication

Biofilm-associated infections are difficult to treat because bacteria are protected by an extracellular matrix and often have low metabolic activity. Phages can disrupt biofilms by infecting susceptible bacteria, while some phages also carry or encode depolymerases that degrade capsular or extracellular polysaccharides. By degrading the biofilm matrix, these enzymes may expose bacterial receptors and improve the access of phages, antibiotics, and host immune defenses to embedded bacteria [20]. Engineered phages expressing biofilm-degrading enzymes have also shown enhanced antibiofilm activity in experimental models.

However, phage penetration does not necessarily result in complete biofilm removal. Treatment efficacy depends on the phage–host combination, biofilm structure and maturity, receptor availability, dose, and treatment duration. Slow-growing or dormant bacteria, receptor masking, limited access to protected bacterial populations, and phage resistance may reduce phage replication and bacterial killing. Phage cocktails or combinations of phages with antibiotics or depolymerases may improve biofilm disruption, although their effects depend on the selected agents and treatment conditions. Phages should therefore be considered part of a combined treatment strategy rather than a universally effective solution for biofilm eradication.

#### Inherent biocompatibility and safety profile

Bacteriophages depend on bacterial cellular machinery for replication. They cannot productively infect or replicate in human eukaryotic cells. Appropriately selected and purified phage preparations have generally shown good tolerability in preclinical studies and early clinical applications [14]. However, the inability of phages to replicate in human cells does not guarantee complete safety. The safety of phage therapy also depends on phage selection, genomic characteristics, manufacturing quality, purification efficiency, administration route, dose, treatment duration, and patient-specific factors. Therapeutic candidates should be screened to exclude temperate phages and phages carrying lysogeny-associated, toxin, virulence, or antimicrobial-resistance genes. Bacterial endotoxins, host-cell proteins, nucleic acids, and other residual materials must also be removed during production, especially from preparations intended for systemic administration.

Phages can also interact with the human immune system. After systemic or repeated administration, phages may be cleared by phagocytic cells in the liver, spleen, and other tissues, reducing their availability at the infection site. Pre-existing or treatment-induced anti-phage antibodies may also neutralize therapeutic phages, particularly during prolonged or repeated treatment. However, the strength and clinical importance of this response vary among phages and patients. Rapid bacterial lysis may release endotoxins and other inflammatory bacterial components. The resulting response may vary with the infection model and treatment conditions.

## Emerging trends of phage therapy

### Genetic and synthetic bioengineering of phage

The inherent limitations of natural bacteriophages, such as narrow host ranges, potential carriage of virulence genes, and incomplete genetic characterization, constrain their clinical applications. Consequently, modern phage therapy is undergoing a transition from the use of “natural phages” toward the development of “designer phages.” Advances in genetic engineering and synthetic biology now enable targeted modifications to overcome these shortcomings and expand therapeutic utility.

A primary goal is to convert temperate phages into obligately lytic, safer variants by eliminating genes responsible for lysogeny and virulence. Previous studies have engineered temperate phages into obligately lytic forms through the deletion of integrase or repressor genes, yielding therapeutic candidates [21]. The engineering workflow typically involves two phases: Build (genome editing) and Select/Recover (mutant isolation).

Editing relies on efficient recombination systems. For mycobacteriophages, the Bacteriophage Recombineering of Electroporated DNA (BRED) method is a standard approach. BRED involves electroporating donor DNA into phage-infected cells, where host or phage recombinases facilitate homologous recombination. Its high efficiency often allows mutant identification through PCR screening of only a small number of plaques. A major challenge is the inability to use antibiotic resistance markers for phage selection. While traditional methods like plaque screening are inefficient, CRISPR-Cas systems offer a powerful solution [22]. By expressing Cas9/Cas12 with guide RNAs targeting the wild-type phage genome in the host, the system selectively degrades parental genomes, enriching for recombinant progeny. When combined with BRED, this strategy forms the CRISPY-BRED platform, which dramatically improves the recovery of low-efficiency recombinants such as fluorescent reporter phages. Furthermore, recent studies have developed RNA-targeting CRISPR-Cas13a systems, which enable complex edits including single-codon deletions and the insertion of fluorescent tags into jumbo phages [23].

Beyond intracellular editing, synthetic biology enables *de novo* phage construction. Complete phage genomes (e.g., T7, φX174) have been synthesized and “booted” in cell-free systems, demonstrating the feasibility of bottom-up assembly [24]. This approach holds promise for future on-demand manufacturing platforms, in which phages could be rapidly synthesized at clinical sites based on the characteristics of the infecting pathogen.

Furthermore, engineering strategies facilitate diverse functional enhancements, such as broadened host range through altering the receptor-binding domains of tail fiber proteins; enhanced lytic activity by upregulating expression of endolysins, biofilm penetration by encoding polysaccharide-degrading enzymes and immune evasion by modifying capsid proteins via PEGylation or targeted mutagenesis to reduce immune recognition [25].

### Phage-derived proteins

Phage-derived proteins represent a cornerstone of phage-based therapeutic strategies, offering a versatile arsenal of naturally evolved antibacterial agents.

#### Endolysins

Endolysins are phage-encoded hydrolytic enzymes that degrade the bacterial peptidoglycan (PG) layer from within, facilitating the final lysis of the host cell to release progeny virions [26]. Their exceptional catalytic efficiency leads to rapid osmotic lysis and bacterial death. Since the earliest application of a purified lysin against *Streptococcus pyogenes* in 1959, numerous endolysins have been identified and evaluated [27]. Studies consistently show that endolysins exhibit rapid and potent bacteriolytic activity against Gram-positive bacteria such as *Staphylococcus*, *Streptococcus*, and *Bacillus*, and have shown efficacy in various animal infection models.

Gram-positive phage endolysins typically feature a modular architecture with an enzymatically active domain (EAD) that is responsible for cleaving specific PG bonds, and a cell wall-binding domain (CWBD), which recognizes defined epitopes on the bacterial cell wall, connected by a flexible linker [28]. EADs can target various chemical linkages within PG, including β-1,4 glycosidic bonds, MurNAc-L-Ala amide bonds, and peptide crosslinks, ultimately leading to mechanical imbalance and instantaneous lysis [29]. While the outer membrane of Gram-negative bacteria poses a barrier, engineering strategies and the discovery of endolysins with intrinsic membrane-permeabilizing activity have enabled effective targeting of critical Gram-negative pathogens like *Acinetobacter baumannii*, *Klebsiella pneumoniae*, and *Pseudomonas aeruginosa* [30].

#### Depolymerases

Depolymerases are essential phage-encoded enzymes that dismantle the external polysaccharide barriers of bacteria, including protective capsules and biofilm matrix [16]. Typically located on tail fibers or spikes, these enzymes hydrolyze specific glycosidic linkages within the extracellular polymeric substances (EPS) [31]. They can facilitate phage infection against pathogens with thick capsules such as *K. pneumoniae* and *A. baumannii* by degrading capsules to expose cellular receptors. Purified depolymerases can be used as standalone enzymatic therapeutics capable of rapidly disrupting mature biofilms. By degrading the biofilm matrix, depolymerases revert embedded bacteria to a planktonic state, thereby dramatically enhancing the efficacy of co-administered antibiotics and host immune clearance [31]. As such, depolymerases are widely regarded as powerful antimicrobial adjuvants that synergistically dismantle the most robust defensive layers of superbugs.

#### Anti-CRISPR proteins

A more recent discovery is the family of phage-encoded anti-CRISPR (Acr) proteins, which inhibit bacterial CRISPR-Cas adaptive immune systems. Their existence was first inferred from the susceptibility of CRISPR-Cas-containing *P. aeruginosa* lysogens to phage infection, suggesting prophage-encoded suppression of host immunity [32]. Structural and biochemical investigations have shown that Acr proteins block key steps of CRISPR-Cas immunity through diverse mechanisms. Some Acr proteins stoichiometrically or enzymatically block target recognition or nuclease activity. A study identified an Acr called AcrIF25 and showed that it binds the Cas7 subunit of the Type I-F Csy complex. This interaction disintegrates Cas7 from the CRISPR RNA (crRNA), thereby pulling apart the entire functional complex [33]. Another instance is AcrIIC3, which induces aberrant dimerization of Cas9, preventing functional complex formation [34]. AcrIF9 induces non-specific DNA binding by the Csy/Cascade complex, diverting Cas proteins from their true phage DNA targets and rendering the immune system ineffective. Other Acr proteins can mimic defective Cas proteins or crRNAs. By incorporating into Cas complexes, they disrupt their structural or functional integrity through dominant-negative effects [35].

### Phage-antibiotic synergy

Phage-antibiotic synergy (PAS) has emerged as a promising strategy to combat MDR pathogens [36]. PAS refers to the phenomenon where the combined application of phages and antibiotics yields a bactericidal effect exceeding the sum of their individual activities. This synergy can more effectively reduce bacterial burden and suppress the emergence of resistance to either agent. The outcome of PAS is highly contingent on factors such as antibiotic class, dosing sequence, concentration, and bacterial physiology. Notably, cell wall synthesis inhibitors and DNA-damaging agents most frequently demonstrate synergy, while protein synthesis inhibitors often show neutral or antagonistic effects [37].

The primary mechanism of traditional PAS involves antibiotic-induced alterations to bacterial morphology or physiology that enhance phage infectivity and productivity. For example, β-lactams and quinolones can cause cellular filamentation and increase cell surface area, which promotes phage adsorption. Certain antibiotics can enhance host metabolic activity or prolong the phage lytic cycle, resulting in a larger burst size. In addition, weakening the cell wall can lower the lysis threshold, allowing more complete phage-mediated lysis. Multiple studies across *E. coli*, *P. aeruginosa*, *Burkholderia cenocepacia*, and others have shown that sub-inhibitory antibiotic concentrations can enlarge phage plaques, elevate phage titers, and accelerate lysis [38].

Recent research has expanded the PAS paradigm through the concept of temperate PAS (tPAS), where temperate phages contribute to synergy under specific antibiotic pressure. Al-Anany and colleagues first introduced this term after observing over 8-log bacterial clearance when combining the temperate *E. coli* phage HK97 with ciprofloxacin [39]. Unlike traditional PAS, tPAS is not driven by antibiotic-enhanced phage replication. Instead, fluoroquinolone-induced DNA damage activates the SOS response and synchronously induces prophages integrated in the bacterial chromosome, leading to rapid lysis of lysogenic cells. More importantly, ciprofloxacin selectively inhibits lysogens carrying prophages, making them a metabolic burden within the population and enabling rapid population-level clearance. It should be noted that this DNA damage-dependent, SOS-driven induction mechanism aligns closely with classical findings. As early as 1959, Otsuji and colleagues reported that mitomycin C could effectively induce prophages in lysogenic *E*. *coli* K-12 into the lytic cycle, establishing the foundational concept that antibiotics can function as triggers for prophage induction [40].

Beyond immediate killing, PAS can critically alter bacterial evolutionary trajectories under dual selection pressure. Under phage-only pressure, resistance often arises via low-fitness-cost receptor modifications (e.g., *wcaJ* mutations in *K. pneumoniae*) [41]. In contrast, PAS conditions with β-lactams can select for high-cost metabolic defects (e.g., *galU* mutations) that impair bacterial growth and fitness [42]. This “evolutionary rerouting” toward less fit genotypes demonstrates how PAS can constrain the emergence of stable, robust resistance lineages.

PAS has shown significant potential against multiple Gram-negative pathogens, including *P. aeruginosa*, *A. baumannii*, *K. pneumoniae*, and *Burkholderia spp.*, enhancing killing, overcoming resistance, and promoting biofilm clearance. However, synergy is not universal and depends heavily on specific phage-antibiotic-pathogen combinations. Under simulated physiological conditions (e.g., urine, serum), reduced bacterial metabolic activity and slower proliferation can weaken phage replication, diminishing synergy. Additionally, certain antibiotics may inhibit critical steps of phage infection at specific concentrations, leading to antagonism [38].

## Phage therapy workflow: from bench to bedside

### Phage sourcing and biobanking

Phages are the most abundant biological entities on Earth, with an estimated global population of 10³¹ particles and a collective biomass of approximately 200 million tons [43]. They are ubiquitous across virtually every explored biome, from the human gastrointestinal tract and soil to aquatic and extreme environments [44]. This immense environmental reservoir represents a foundational resource for therapeutic discovery. The choice of sourcing environment is strategic, as viral composition varies significantly across geographical and ecological niches [45]. Oceans and freshwater systems are prolific phage habitats, and tailed phages historically classified within the *Myoviridae* family are frequently isolated from these sources. Sewage, which concentrates microbial communities from human and animal waste, is a particularly rich and clinically relevant source for isolating phages that target human-associated bacteria. The isolation strategy is tailored to the phage life cycle and sample type. Virulent phages can be directly isolated from diverse natural environments, while temperate phages require induction to enter the lytic cycle.

Several globally recognized repositories provide essential phage strains for research and development, such as Leibniz Institute DSMZ and the Adaptive Phage Therapy Phage Bank (APT), which curate diverse phages targeting multiple bacterial hosts [5]. Complementing these physical collections are digital genomic biobanks. The curated PhageScope database includes 873,718 phage sequences and the Actinobacteriophage Database at phageDB includes more than 5000 mycobacteriophages [46]. These resources catalog phage genomes and their predicted host ranges, enabling in silico identification of candidate phages against specific pathogens.

Advances in synthetic biology are revolutionizing phage sourcing. The *de novo* synthesis of complete phage genomes from digital blueprints and their “rebooting” in cell-free systems now allow for the creation of phages without natural isolation, enabling rapid production and engineering of variants [47].

### Phage characterization and selection

#### Host range determination

A phage’s host range is a fundamental property governing its therapeutic utility. Traditional phenotypic methods, primarily the plaque assay (PA) and its variants (e.g., double-layer agar, spot test), remain widely used for host range profiling [48]. These methods visually confirm lytic activity through plaque formation but are labor-intensive, time-consuming, and limited to cultivable host strains.

As the traditional phage plaque assay is time-consuming and not conducive to rapid diagnosis, a variety of rapid screening methods have been developed to detect phage infection directly or indirectly. Real-time quantitative PCR (qPCR) can detect infection by measuring an increase in phage concentration; however, it requires the design of specific primers and the optimization of conditions for each phage, and is therefore not suitable for high-throughput screening of large phage libraries [49]. Flow cytometry rapidly identifies susceptible hosts by detecting physiological changes in bacteria following phage infection, enabling analysis of phage–host interactions within hours. However, it requires specialized equipment and optimization for different phage–host systems. Virus labeling techniques identify the host by fluorescently labeling phages and combining this with flow cytometric sorting; however, they can only detect phage attachment and cannot determine whether effective infection and lysis have occurred [50]. Furthermore, bioluminescence or colorimetric detection of endogenous enzymes released during cell lysis can indirectly indicate phage proliferation. A study integrated ATP-based bioluminescence technology into a freeze-dried high-throughput platform, enabling the rapid screening of therapeutic phages within 30 to 120 min [51].

Recent advances in sequencing technology enable the discovery and identification of phages and their hosts from environmental samples, providing a crucial pathway for comprehensive studies of natural viral diversity [52]. These leverage genomic signatures and co-evolutionary patterns identified through bioinformatics analysis of sequencing data. Prediction strategies fall into three main categories: alignment-based approaches, which identify homologous sequences shared between phages and bacterial hosts; alignment-free methods, which compare sequence composition patterns [53]; and machine learning approaches, which utilize features like nucleotide content, protein domains, and co-abundance patterns from metagenomic data to infer host ranges. While powerful for screening vast sequence datasets, computational predictions require phenotypic validation. An integrated strategy, combining high-throughput in silico screening with subsequent in vitro confirmation, represents a robust and efficient path forward for characterizing phage host ranges.

#### Genomic sequencing and analysis

The field has moved far beyond the first phage genome sequenced in 1977, propelled by next-generation and long-read sequencing technologies [54]. Viral metagenomics, in particular, has been transformative. This cultivation-independent approach enables the direct, high-throughput sequencing of viral communities, revealing the immense and previously unseen genetic diversity of phages. These advances have cataloged tens of thousands of complete phage genomes, highlighting extraordinary variation in genome size, architecture, and nucleic acid composition. Another powerful, though more resource-intensive, tool is single-cell genomics. By isolating and sequencing individual microbial cells, this method can concurrently capture the genome of a host bacterium and any infecting phage, providing direct insights into natural infection states [55]. Together, these sequencing strategies form the foundation for the safe and informed selection of phages, ensuring that candidates are genetically suited for clinical applications.

#### In vitro efficacy

Demonstrating potent lytic activity against the target pathogen under controlled conditions is a prerequisite for therapeutic development. In vitro efficacy testing is typically tiered into preliminary screening and confirmatory quantitative assays [56]. Preliminary screening methods allow rapid evaluation of many phage-bacterium pairs, such as spot tests and liquid culture lysis. Spot tests involve applying phage lysate drops onto a bacterial lawn to observe zones of clearing, which is simple and economical. Liquid culture lysis monitors bacterial growth inhibition in broth using optical density, which is especially useful for strains that do not form clear plaques [57].

On the other hand, confirmatory quantitative assays provide precise metrics of phage potency. The plaque assay is the gold standard, used to determine the phage titer (plaque-forming units per mL, PFU/mL) and evaluate efficiency of plating (EOP). Methodological variations, such as testing single versus multiple dilutions, balance throughput with precision [56].

### Phage formulation and delivery

#### Purification and safety

A paramount challenge in therapeutic phage preparation is the separation of phages from bacterial debris and the removal of endotoxins, which pose significant safety risks. While cesium chloride density gradient ultracentrifugation remains a gold standard at the laboratory scale for achieving high purity, its scalability is limited, and it involves hazardous materials unsuitable for large-scale pharmaceutical production [58]. Consequently, research focuses on developing scalable, Good Manufacturing Practice (GMP)-compliant purification pipelines.

Hietala V et al. compared the feasibility of removing endotoxins and protein toxins during clinical phage preparation using various purification methods including PEG precipitation, sucrose gradient ultracentrifugation, and anion exchange chromatography. They found that combining affinity column chromatography with ultrafiltration yielded the optimal phage preparation [58]. Saavedra JPP et al. proposed a scalable purification strategy combining alkaline phosphatase treatment with anion exchange chromatography, and ultimately achieved a phage titer of up to 1.26 × 10^11^ PFU/mL with endotoxin removal rates increased from 87.3% to 98.8% [59]. Luong T et al. outlined a systematic and practical phage concentration and purification workflow, which utilizes tandem capsule filtration, cross-flow ultrafiltration concentration combined with dialysis to remove small-molecule impurities such as endotoxins and exotoxins [60].

To summarize, preliminary purification typically involves the removal host cells and cell debris via centrifugation and filtration, followed by more selective operations such as ultrafiltration, polyethylene glycol (PEG) precipitation, and chromatography techniques to concentrate phages and remove bulk contaminants. More importantly, dedicated steps such as lipopolysaccharide affinity chromatography, alkaline phosphatase treatment, or optimized tangential flow filtration are critical to reduce endotoxin levels to permissible thresholds for clinical use [60].

#### Formulation and delivery routes

**Fig. 4:**
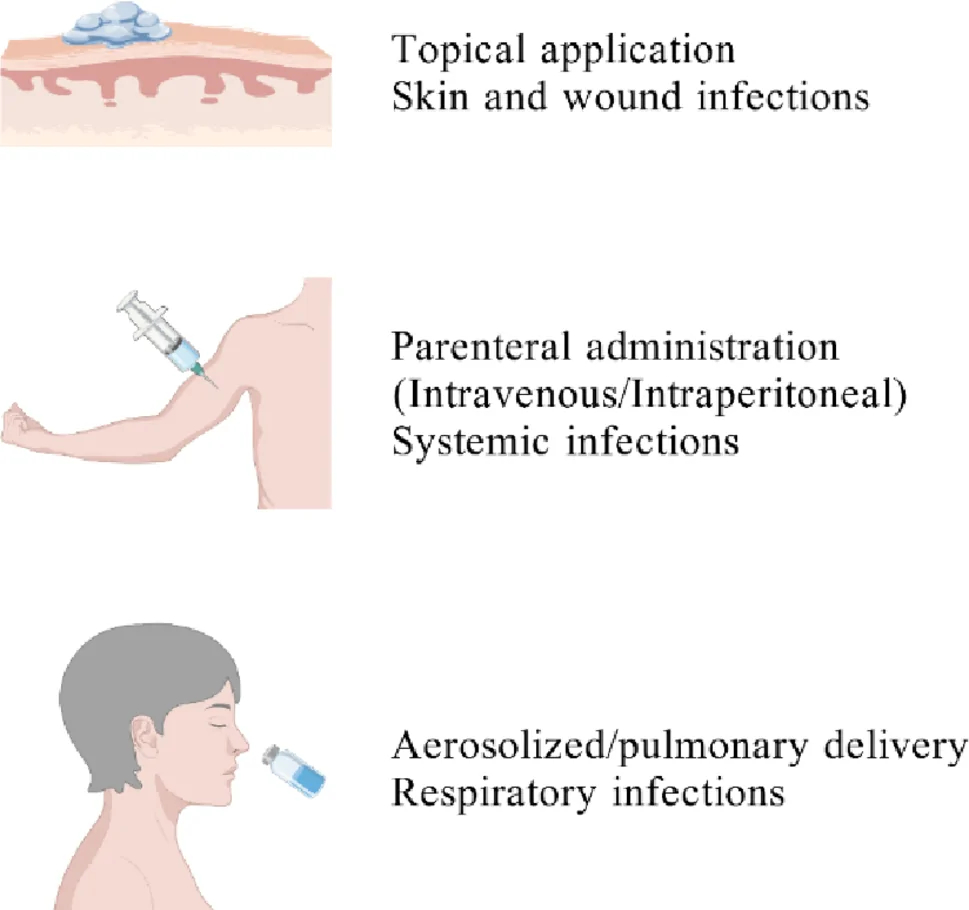
Methods of phage administration

The choice of formulation is dictated by the intended route of administration, which is selected based on the infection site (Fig. 4).

For skin and wound infections, phages are incorporated into topical vehicles such as hydrogels, creams, or ointments. Hydrogels, in particular, provide a moist wound environment, sustain phage release, and maintain phage viability at the infection site, promoting both bacterial clearance and wound healing .

For systemic or deep-seated infections, phages are formulated in sterile buffered solutions like phosphate-buffered saline (PBS) and Tris-HCl-buffered magnesium chloride (SMB) [61]. Intravenous (IV) infusion allows phages to circulate and reach internal infection sites, though rapid clearance by the reticuloendothelial system can limit bioavailability. Intraperitoneal (IP) injection can achieve higher initial concentrations and longer circulation times, facilitating delivery to organs like the lungs and spleen.

To treat respiratory infections, phages are engineered into inhalable dry powder formulations, which deliver high local concentrations directly to the lungs. Spray drying is a common method, but the associated heat and shear stress can inactivate phages. To overcome this, stabilizing excipients are used. Yan et al. incorporated an anti-*A. baumannii* phage into a dry powder formulation containing trehalose, mannitol, and L-leucine, thereby reducing particle aggregation [62]. Carrigy et al. developed a phage powder spray-drying and packaging process utilizing a dual-fluid atomizer with leucine and trehalose as excipients, producing a dry powder of anti*-Campylobacter jejuni* phage suitable for global distribution without cold chain infrastructure [63]. Pathak V et al. investigated a composite spray-dried matrix comprising hydrolyzed gelatin as stabilizer and leucine or trimethylalanine as dispersion enhancers, achieving high phage stability and excellent atomization performance for treating pulmonary bacterial infections [64].

A critical consideration across all routes is the potential for the host immune system to neutralize phage particles, which may impact the pharmacokinetics and efficacy of repeat dosing. Therefore, ongoing pharmacodynamic (PD) and pharmacokinetic (PK) studies are essential to optimize dosing regimens.

#### Cocktail design

Employing a cocktail of multiple phages, rather than a single isolate, is a strategic approach to enhance therapy. It broadens the spectrum of activity against a bacterial species and critically reduces the rate at which bacteria evolve resistance [65]. For clinical applications, two approaches exist for preparing phage cocktails: “ready-to-use” and “customized”. The ready-to-use approach employs pre-formulated fixed mixtures targeting common high-priority pathogens [66]. This approach enables rapid deployment and simplified regulatory and manufacturing pathways but may lack precision for rare strains. The personalized approach involves tailoring a cocktail specifically to the susceptibility profile of the bacterial isolate obtained from an individual patient. This approach promises higher precision and can combat pre-existing resistance but is more time-consuming and resource-intensive.

The rational design of an effective cocktail is guided by several core principles to ensure safety, potency, and durability. Phages should utilize distinct bacterial receptors for adsorption to minimize the potential for cross-resistance and work cooperatively, for instance, through complementary mechanisms where one phage encodes depolymerases that expose receptors for another. Besides, all component phages should be strictly lytic and genetically safe, and the final formulation should be stable.

Rationally designed phage cocktails constitute a powerful and increasingly validated therapeutic strategy. Development efforts are particularly advanced for cocktails targeting ESKAPE pathogens (*Enterococcus faecium*, *Staphylococcus aureus*, *K. pneumoniae*, *A. baumannii*, *P. aeruginosa*, and *Enterobacter* spp.), with empirical efficacy demonstrated across in vitro, biofilm, and in vivo infection models [65].

### Key therapeutic areas

Phage therapy is being actively investigated across a spectrum of challenging clinical infections where conventional antibiotics often fail, particularly those involving MDR pathogens, biofilms, or compromised anatomical sites.

#### Complex chronic infections

Chronic, refractory infections such as diabetic foot ulcers and osteomyelitis represent a major therapeutic challenge due to biofilm formation, poor antibiotic penetration, and MDR pathogens. Phage therapy has shown utility as an adjuvant in these settings. For instance, topical application of the *S. aureus* phage Sb-1 successfully resolved a complex diabetic toe ulcer, preventing amputation [67]. Similarly, phage therapy has been employed in cases of chronic bacterial prostatitis (CBP) caused by polymicrobial, antibiotic-resistant biofilms, where a combination of oral, rectal, and urethral phage delivery eradicated pathogens that had persisted despite prolonged antibiotic courses [68]. These cases underscore phage therapy’s potential to overcome anatomical and resistance barriers, though its efficacy can be limited in polymicrobial infections if the available phage library lacks coverage for all causative agents.

#### Cystic fibrosis and pulmonary infections

The airways of cystic fibrosis (CF) patients are chronically colonized by resilient bacterial biofilms, primarily *P. aeruginosa* and *S. aureus*, which are frequently MDR [69]. Phage therapy is being explored to reduce bacterial load and inflammatory damage. Preclinical studies in CF caused by *P. aeruginosa* demonstrate that phage treatment can significantly decrease mortality, bacterial burden, and pro-inflammatory cytokines [70]. Beyond *P. aeruginosa*, compassionate use cases have reported clinical improvements in CF patients with infections caused by *S. aureus*, *Achromobacter*, *Burkholderia* or *Mycobacterium abscessus*, supporting further investigation into inhaled phage formulations for respiratory delivery [71].

#### Burns and wound infections

Burn wounds and other severe skin injuries are highly susceptible to infection by MDR pathogens like *P. aeruginosa*, *A. baumannii*, and *S. aureus*, which can lead to sepsis. Topical phage delivery via thermosensitive hydrogels loaded with bacteriophages against *A. baumannii* allows for sustained, high-concentration application directly to the wound site, promoting healing and preventing systemic spread [72]. McVay et al. demonstrated that a single dose of *P. aeruginosa* phage cocktail significantly reduced mortality in a mouse burn model, achieving an 87% survival rate post-treatment [73]. Chadha et al. have shown that both monophage and cocktail therapies can significantly improve survival rates and reduce bacterial counts in mice with burn wound infections caused by *K. pneumoniae* [74]. However, optimal dosing strategies remain an area of active research, as studies have reported variable efficacy with different phage concentrations.

#### Systemic and resistant infections

For life-threatening systemic infections like bacteremia and sepsis, the rapid, bactericidal action of phages is particularly valuable. Studies in murine models have shown that phage cocktails can rescue animals from lethal bacteremia caused by MDR *E. coli* or *K. pneumoniae*, even when treatment is delayed [75]. Furthermore, phages exhibit dynamic adaptability. Salazar et al. demonstrated that phages can evolve to infect resistant bacterial variants that emerge during treatment, a feature that underscores their potential for managing evolving infections. Another study observed significant phage replication in diverse tissues, decreased bacterial counts, as well as levels of pro-inflammatory cytokines like IL-6 after administration of phages in a *S. aureus* sepsis model [76].

#### Gastrointestinal and foodborne pathogens

Phage therapy offers a targeted approach to gastrointestinal infections, aiming to eradicate pathogens like *E. coli*, *Salmonella*, and *Clostridium difficile* while sparing the commensal microbiota. Oral phage therapy (OPT) is the primary route, though phage viability must be protected from gastric acidity through encapsulation technologies [77]. Advancements in encapsulation technology have enhanced phage stability during passage through the acidic gastric environment, bolstering the potential of OPT. A study demonstrated that a phage cocktail reduced *E. coli* colonization in the gut and improved outcomes in animal models [78]. Beyond therapeutics, phages have significant biocontrol applications in the food industry. They are approved as food additives to reduce contamination by pathogens such as *Listeria monocytogenes* on ready-to-eat meats and are used to target spoilage organisms, thereby enhancing food safety and extending shelf life [79].

### PK/PD modeling

Although bacteriophages have attracted considerable attention as an alternative to antibiotics, their ability to self-replicate makes their in vivo pharmacokinetics highly complex in the presence of susceptible bacteria. Phage biodistribution is influenced by the route of administration, phage characteristics, the site of infection, and host immune status. After administration, phages can distribute to the bloodstream and multiple tissues, with the liver, spleen, and lungs rich in the mononuclear phagocyte system, typically showing the highest accumulation [80]. Different administration routes also affect local phage exposure, with nebulization improving delivery to the lungs and local administration increasing phage concentrations at infection sites. Phage clearance is primarily mediated by the host immune system through macrophage phagocytosis, complement activation, and neutralizing antibodies, resulting in marked differences in half-life and clearance among phages. Following intravenous administration, phage titers generally peak within 1–3 h and decline to near-baseline levels within 24–72 h. In addition, phage concentrations are determined not only by host-mediated clearance but also by interactions with susceptible bacteria. Factors including bacterial density, adsorption rate, latent period, and burst size influence phage replication, giving rise to infection- and density-dependent pharmacokinetic profiles [81]. Accordingly, current PK/PD models increasingly integrate phage biodistribution, immune clearance, and phage–bacteria dynamics to optimize dosing strategies and improve therapeutic efficacy. Nevertheless, more comprehensive dynamic models are still needed to support the clinical translation of phage therapy.

### In vivo phage resistance evolution

The development of phage resistance within the host is a major challenge limiting the long-term efficacy of phage therapy. Under the selective pressure imposed by phage treatment, bacteria can rapidly evolve resistance through multiple mechanisms. The most common mechanism involves modification or loss of phage receptors, including lipopolysaccharides (LPS), capsular polysaccharides(CPS), outer membrane proteins, fimbriae, and flagella, thereby preventing phage adsorption. In addition, intracellular defense systems, such as restriction–modification (R-M), CRISPR-Cas, and abortive infection (Abi), can recognize and eliminate phage nucleic acids. In vivo, resistance evolution is further shaped by host immunity, the infection microenvironment, and bacterial population heterogeneity, making it more complex than in vitro. Importantly, phage resistance often incurs fitness costs, including reduced virulence, impaired biofilm formation, decreased motility, and increased susceptibility to antibiotics or host immunity [82]. Exploiting these trade-offs through phage cocktails, phage–antibiotic combination therapy, and phage–bacteria co-evolution strategies may help delay resistance development and improve the long-term efficacy of phage therapy.

## Clinical case analysis

### Compassionate use cases

Compassionate use involves administering investigational therapies outside of clinical trials to patients with life-threatening conditions who have exhausted all approved treatment options. Given the critical unmet need in treating MDR infections, bacteriophage therapy has emerged as a viable option for compassionate use due to its historical precedent, supportive preclinical data, and favorable safety profile [83]. Published case reports indicate that compassionate phage therapy is most frequently applied against infections caused by *S. aureus*, *P. aeruginosa* and *E. coli*. These cases span a wide range of acute and chronic conditions, including osteoarticular, respiratory, wound, and systemic infections, utilizing various administration routes (topical, intravenous, inhaled). Clinical outcomes from these individual cases, while observational, generally report positive results, including pathogen clearance and symptom improvement [83]. A summary of selected compassionate use cases is provided in Table 1.

However, interpreting this body of evidence requires caution. The heterogeneity in pathogens, infection types, treatment protocols, and outcome measures across different reports makes direct comparisons and definitive conclusions challenging. Publication bias may also lead to an over-reporting of successful cases, whilst cases of treatment failure or inconclusive outcomes are under-reported. Consequently, although compassionate use has yielded valuable clinical experience, its findings cannot replace the evidence provided by rigorously designed randomized controlled trials (RCTs), which remain crucial for establishing the efficacy of phage therapy and determining its optimal application.

**Table 1:** Representative compassionate-use cases

Pathogens	Site of infection	Route of administration	Number of patients	Outcomes	Reference
*A. baumannii*	Pulmonary	Nebulization	4	Two patients successfully treated; one patient died from a subsequent carbapenem-resistant *K. pneumoniae* infection; one died from respiratory failure post-discharge.	[84]
*K. pneumoniae*	Pulmonary	Inhalation; Nasogastric tube	1	Pathogen cleared from bronchoalveolar lavage fluid after one month; no serious adverse events over a 3-year follow-up.	[85]
*P. aeruginosa*	Pulmonary	Nebulization	9	Median reduction in bacterial load in sputum	[86]
	Multiple^a^	Intravenous injection	15	6 cured; 7 experienced symptom relief; 2 deaths occurred in patients with severe comorbidities and MDR infections.	[87]
*S. aureus*	Wounds	Topical	6	Treatment successful.	[67]
	Pleural cavity	Intrapleural	1	S. aureus eradicated, but patient ultimately died due to surgical graft failure.	[85]
Mixed^b^	Skin	Topical	32	16 cured; 7 markedly improved; 2 transiently improved; 7 abandoned.	[88]
*S. aureus; S. pyogenes*	Prostate	Oral; Rectal; Urethral	1	Treatment successful.	[68]

a, Bone, joint and prosthesis-related infections; Bacteremia; Skin and soft tissue infections; Otitis media; Sinusitis

b, *Pseudomonas* spp.; *Staphylococcus* spp.; *Klebsiella* spp.; *Proteus* spp.; *Escherichia* spp

### Clinical trials

While numerous compelling case reports from compassionate use have fueled optimism for phage therapy, results from controlled clinical trials have yielded more heterogeneous outcomes, highlighting the challenges of translating anecdotal success into statistically robust evidence (Table 2).

**Table 2:** Clinical outcomes summary from major phage therapy studies

Patient population	Number of patients	Treatment approach	Clinical improvement	Bacterial eradication	Key findings	References
*P. aeruginosa* burn infections	27	phage PP1131	No significant benefit	Slower than control	Trial terminated for insufficient efficacy	[89]
Device-related / systemic infections	12	Customized phage cocktails	58%	42%	Some occurred immune neutralization or secondary infections.	[90]
Multiple infections	100	Individualized phage therapy	77.2%	61.3%	Effect is reduced by 70% without antibiotics	[91]
Compassionate use cases	> 20	Personalized treatments	70%	50%	Variable outcomes based on infection type	[92]
LVAD infection	4	Intravenous phage therapy	0%	0%	All presented bacteremia	[93]

Several randomized controlled trials (RCTs) have failed to demonstrate clear superiority of phage therapy over standard of care or placebo. For instance, a urology RCT found no significant difference in success rates between phage therapy, antibiotics, and a placebo group, phage treatment was reported to be non-inferior to standard antibiotic therapy but was not superior to placebo bladder irrigation [94]. This finding did not demonstrate an independent therapeutic benefit of the phage preparation and also highlighted the potential influence of the administration procedure and comparator effects on trial outcomes. Similarly, an oral phage cocktail showed no improvement over placebo in treating acute bacterial diarrhea in children, despite a favorable safety profile [95]. Instability in phage preparation and formulation has also been cited as a contributing factor to suboptimal outcomes in some trials.

Conversely, other studies have reported positive results. A double-blind trial in COVID-19 patients with secondary bacterial pneumonia found that nebulized phage therapy significantly improved clinical parameters (e.g., oxygenation, symptom resolution, and hospitalization duration) compared to standard care [96]. Furthermore, a review noted clinical cure in 93.1% of 277 patients with osteoarticular infections [97], and another noted complete or partial cure in 96.8% of 1,307 patients with purulent infections caused by MDR pathogens. However, these favorable estimates were largely derived from heterogeneous observational or historical data and should not be interpreted as equivalent to evidence from well-controlled randomized trials.

Although preclinical studies and compassionate-use cases have highlighted the therapeutic potential of phage therapy, these encouraging findings have not been consistently confirmed in randomized controlled trials (RCTs). Most compassionate-use evidence is derived from uncontrolled case reports or small case series, limiting the ability to assess the independent efficacy of phage therapy. In contrast, several RCTs, including the PhagoBurn trial and studies of phage therapy for urinary tract infections, failed to demonstrate significant superiority over standard treatments, although these trials generally reported favorable tolerability. This discrepancy underscores critical developmental challenges. It emphasizes the necessity of moving beyond anecdotal evidence through well-designed trials that utilize well-characterized, stable phage preparations with optimized dosing regimens. Analyzing both successful and unsuccessful trials is invaluable for identifying the key parameters, such as phage selection, pharmacokinetics, and patient stratification, which are essential for demonstrating consistent therapeutic efficacy. This rigorous, evidence-based approach is fundamental to realizing the full clinical potential of phage therapy.

## Challenges and limitations

### Scientific and technical hurdles

The dynamic biology of phages, while the source of their therapeutic advantage, also presents significant scientific challenges. Just as bacteria evolve resistance to antibiotics, they can rapidly develop resistance to phages through mechanisms such as receptor modification, CRISPR-Cas systems, and abortive infection systems. This necessitates the use of well-characterized phage cocktails or adaptive treatment strategies. Furthermore, the behavior of phages in vivo is complex and poorly standardized. Unlike static drug molecules, phages can amplify at infection sites but may also be neutralized by the host immune system or cleared rapidly by the reticuloendothelial system, making dose prediction difficult [66]. Thus, the phage pharmacokinetics and pharmacodynamics (PK/PD), including their distribution, persistence, replication and clearance should be clarified during the preclinical stage. Most importantly, the narrow host range of most phages is a double-edged sword. Although it minimizes disruption to the commensal microbiome, it complicates treatment when the causative pathogen is not precisely identified or when infections are polybacterial [5]. This often requires the creation of personalized phage cocktails or large, pre-screened phage banks.

### Regulatory challenges

The unique nature of phage therapeutics creates distinct regulatory obstacles. Regulators are accustomed to approving fixed, well-defined drugs for broad patient populations. Many phage applications follow a personalized-medicine paradigm. There is a critical absence of universally accepted standards for key processes like phage isolation, purification, potency titration, and stability testing. This variability complicates quality control, batch consistency, and comparability of clinical data [94]. While regulatory agencies like the U.S. Food and Drug Administration (FDA) and the European Medicines Agency (EMA) are actively developing pathways for phage-based products, the evolving regulatory framework remains more complex and less clearly defined than for conventional antibiotics, adding uncertainty to the development process [92]. Creating a framework that ensures safety and efficacy for both standardized and personalized approaches is a significant regulatory frontier.

### Commercial and manufacturing barriers

The translation of phage science into a viable, scalable industry faces distinct economic and production challenges. Intellectual property (IP) protection is complicated by the natural origin of phages, potentially discouraging investment. Strategies often focus on patenting specific formulations, cocktails, genetic engineering methods, or unique delivery systems rather than the phages themselves. Scaling up GMP production is technologically and financially demanding, particularly for multi-phage cocktails where each component may require unique cultivation and purification processes to ensure high purity and the absence of bacterial toxins. Finally, demonstrating cost-effectiveness and establishing reimbursement models is an unresolved commercial challenge. While phage therapy may reduce long-term costs by shortening hospital stays or curing otherwise untreatable infections, the high upfront development costs and the variable expense of personalized treatments pose significant hurdles for securing payment from healthcare systems.

### Ethical and ecological considerations

Phage therapy also raises ethical and ecological considerations. Personalized or compassionate-use treatment requires clear informed consent because evidence regarding efficacy, optimal dosing, and long-term outcomes remains limited. Equitable access is another concern, as individualized phage screening and preparation are resource-intensive and are mainly available in specialized centers.

The potential effects of phage therapy on microbial community balance have been discussed above. Beyond the patient microbiome, the release and persistence of therapeutic phages may impose selective pressure on environmental bacterial populations and influence the co-evolution of phages and bacteria. Inadequately characterized phages may also carry undesirable genes or contribute to horizontal gene transfer. Therefore, careful genomic screening, environmental risk assessment, and long-term monitoring are needed to support the responsible use of phage therapy.

### Limitations of this review

This review has several limitations. It is a narrative review, and therefore does not provide a quantitative assessment of treatment efficacy. The available evidence is highly heterogeneous in terms of bacterial pathogens, phage preparations, administration routes, treatment regimens, and clinical outcomes. Much of the clinical evidence is derived from case reports, small case series, and compassionate-use studies, which may be affected by publication bias and incomplete reporting of unsuccessful treatments. In addition, phage taxonomy, engineering technologies, manufacturing standards, and clinical evidence are developing rapidly. Therefore, some conclusions may require updating as new experimental and clinical data become available.

## Future perspective

Phage research is being dramatically reshaped by innovative technologies that promise to overcome existing limitations and unlock new therapeutic potential [98]. Two particularly transformative frontiers are the refinement of phage display technology and the integration of artificial intelligence (AI) into phage discovery and design.

Originally developed as a tool for protein-protein interaction studies and antibody discovery, phage display is finding powerful new applications in therapeutic development. It enables the rapid screening of high-affinity peptides or nanobodies that bind to specific bacterial receptors, which can serve as potential therapeutics or be used to broaden the host range of existing phages. Furthermore, identified binding domains can be fused to bactericidal enzymes to create “enzybiotics” that decouple pathogen recognition from the viral life cycle [99]. Engineered phage capsids can be used as nanocarriers to deliver conventional antibiotics, CRISPR-Cas antimicrobial systems, or biofilm-disrupting agents directly to the site of infection, enhancing potency and reducing off-target effects.

The application of AI and machine learning is revolutionizing every stage of the phage therapy pipeline, moving the field from discovery-based to prediction-driven science. AI algorithms can mine vast metagenomic datasets to identify novel phage sequences and predict key therapeutic properties directly from genomic data [100]. This bypasses traditional culturing bottlenecks and streamlines candidate selection. AI can also guide the modification of tail fiber proteins to recognize new bacterial receptors, optimize proteins for enhanced biofilm penetration or immune evasion, and fine-tune lysis kinetics, which promotes precise engineering of phage traits. Ultimately, these capabilities pave the way for the computational design of fully synthetic phages. By intelligently assembling optimal modules for receptor binding, genome delivery, and host lysis, AI can facilitate the creation of purpose-built therapeutic entities from first principles.

The convergence of phage display, AI, and synthetic biology heralds a future where phage-based antimicrobials are not merely selected from nature, but are intelligently designed, multi-functional platforms. These next-generation agents will be precisely targeted, capable of overcoming resistance mechanisms, and amenable to standardized production. By systematically addressing the scientific and technical hurdles, these advanced technological frameworks pave the way for phage therapy to mature into a robust, predictable, and integral component of the global arsenal against antimicrobial resistance.

## Conclusion

Bacteriophage therapy presents a compelling solution to the antimicrobial resistance crisis, leveraging viruses’ natural ability to precisely target and kill bacterial pathogens. Its advantages, including high specificity, self-amplification at infection sites, biofilm penetration, and biocompatibility, are supported by growing clinical evidence from compassionate use and trials. However, significant challenges in pharmacokinetics, bacterial resistance, regulatory pathways, and scalable manufacturing must be systematically addressed. Through continued research, phage therapy is poised to mature into an indispensable, precision component of the global antimicrobial arsenal, offering new hope in the enduring battle against resistant infections.

## Data Availability

The original contributions presented in this study are included in the article. Further inquiries can be directed at the corresponding authors.
